# Supplementary material for: Rapid evolutionary change in trait correlations of single proteins
Source: Nat Commun. 2024 Apr 18;15:3327. doi: 10.1038/s41467-024-46658-1 (PMC11026499; doi:10.1038/s41467-024-46658-1)
Supplement: Supplementary file 1 — Supplementary information [file 41467_2024_46658_MOESM1_ESM.pdf]

Supplementary Information

Rapid evolutionary change in trait correlations of single proteins

Pouria Dasmeh[1,2,3*#], Jia Zheng[2,3,4,8,9*], Ayşe N. Erdoğan[5], Nobuhiko Tokuriki[5], and Andreas Wagner[2,3,6,7#]

[1]Center for Human Genetics, Marburg University, Marburg 35043, Germany.[2]Institute for Evolutionary Biology and Environmental Studies, University of Zurich, Zurich 8057, Switzerland.[3]Swiss Institute of Bioinformatics (SIB), Lausanne 1015, Switzerland. [4]Zhejiang Key Laboratory of Structural Biology, School of Life Sciences, Westlake University; Hangzhou, 310030, China.[5]Michael Smith Laboratories, University of British Columbia, Vancouver, Canada.[6]The Santa Fe Institute, Santa Fe, New Mexico 87501, United States.[7]Stellenbosch Institute for Advanced Study (STIAS), Wallenberg Research Centre at Stellenbosch University, Stellenbosch 7600, South Africa.[8]Westlake Laboratory of Life Sciences and Biomedicine; Hangzhou, 310030, China.[9]Institute of Biology, Westlake Institute for Advanced Study; Hangzhou, 310030, China. (*Equal contribution*)

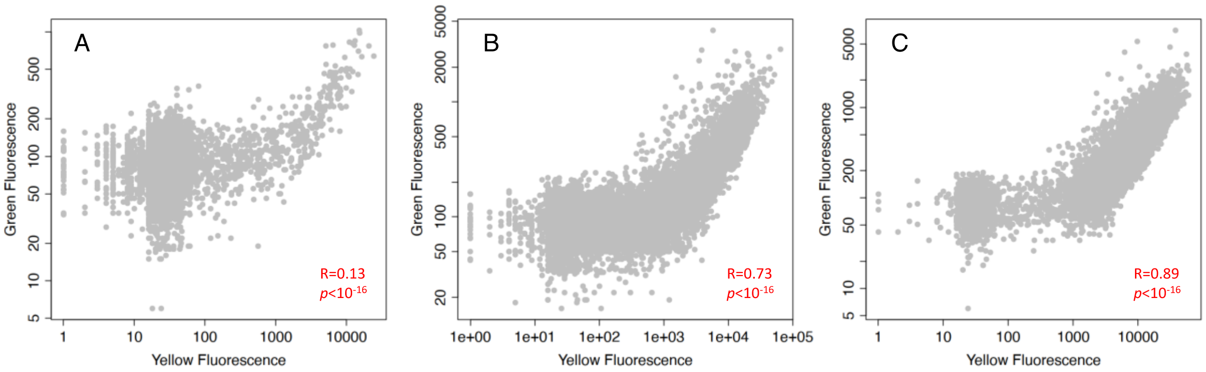

Supplementary Figure 1. Green versus yellow fluorescence intensities for polymorphic populations that had evolved under A) no selection for yellow selection, B) weak selection (top 65 percent survive), and C) strong selection (top 20 percent survive). All correlation coefficients are Spearman's rank correlations.

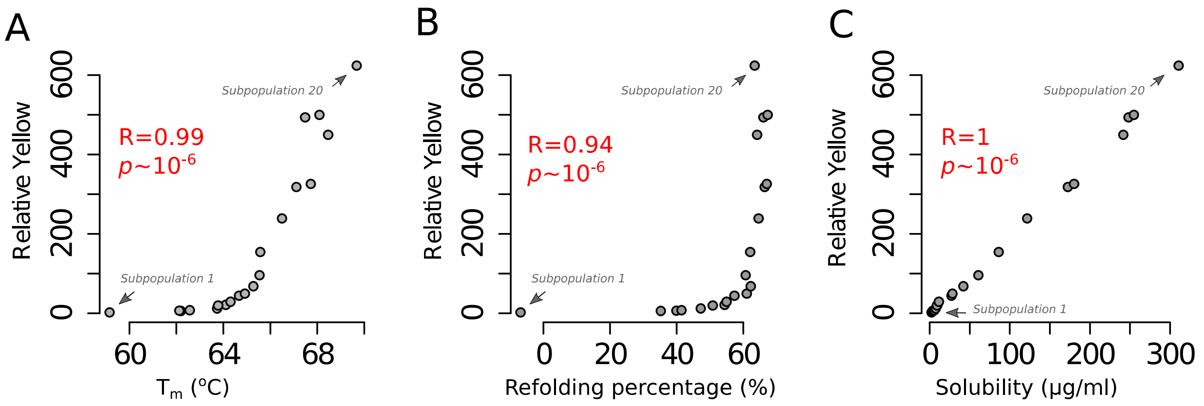

Supplementary Figure 2. Yellow fluorescence intensity (relative to a negative control population) is plotted for all subpopulations (vertical axis) versus A) the temperature of the denaturation midpoint (Tm) of YFP from each subpopulation, B) the refolding percentage upon thermal denaturation, and C) the soluble fraction of YFP proteins (in μg/ml), as assessed by an enzyme-linked immunosorbent assay (ELISA). In panels A-C, subpopulation number increases from the first subpopulation (the left-most circle, labeled), to the 20th subpopulation (right-most circle, labeled). All correlation coefficients are Spearman's rank correlations.

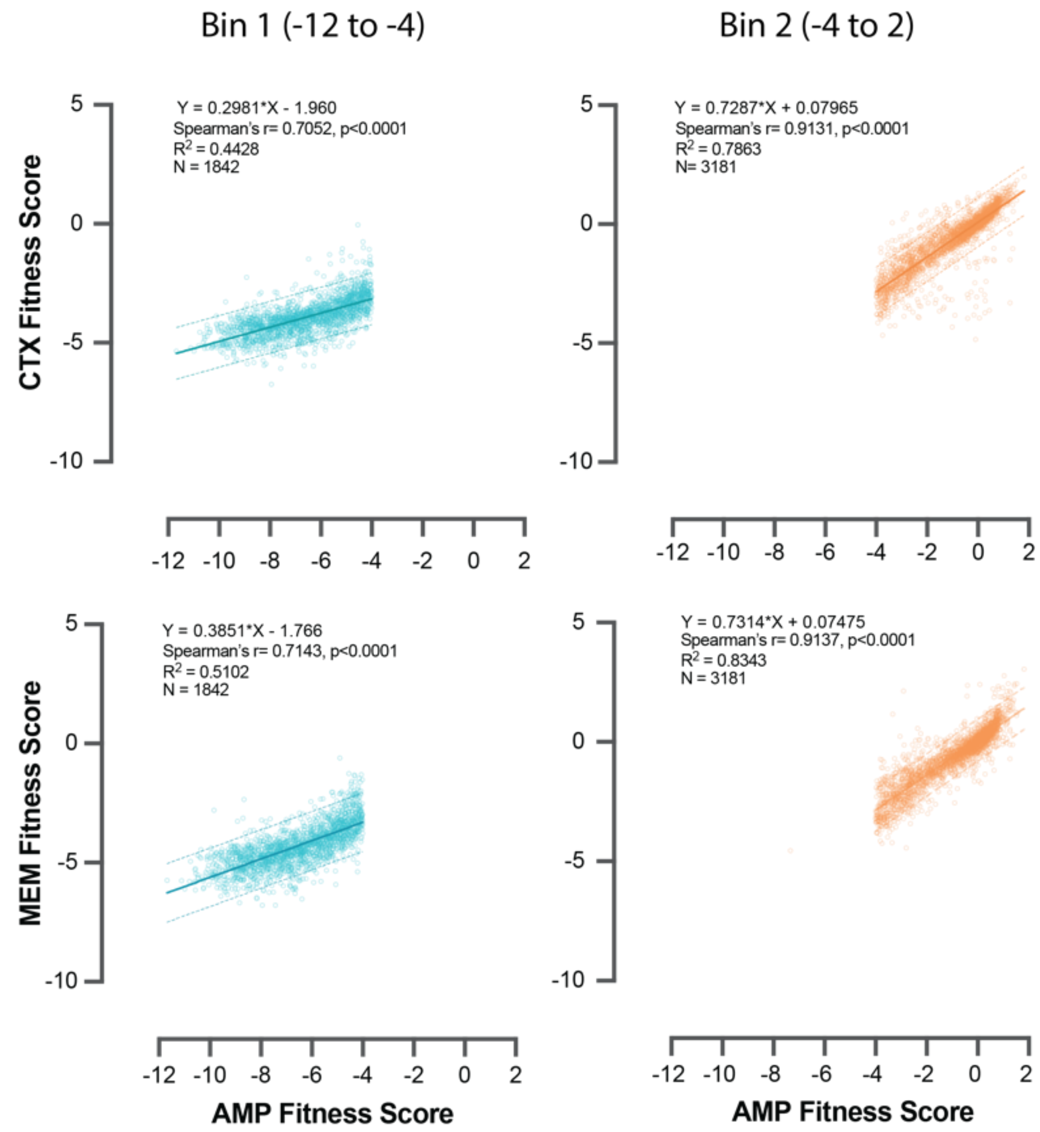

Supplementary Figure 3. Correlation between ampicillin vs. cefotaxime and ampicillin vs. meropenem fitness effects of single point mutations divided into two bins. Data is taken from deep mutational scanning experiments of VIM2 resistance against ampicillin, cefotaxime, and meropenem[1,2].

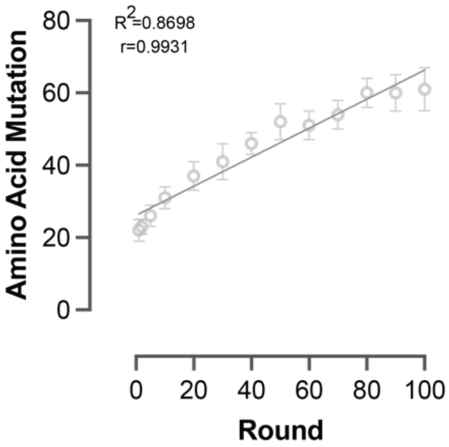

Supplementary Figure 4. The average number of amino acid mutations (accumulated in WT VIM2) in each round of the evolution experiment under an ampicillin concentration of 10 µg/ml. Error bars on the plot represent one standard deviation in the number of amino acid mutations.

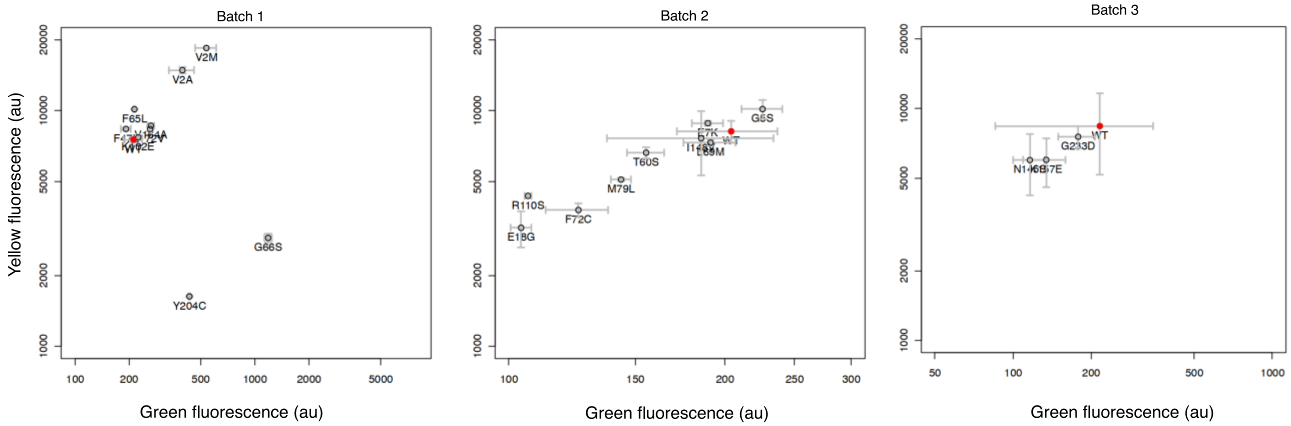

Supplementary Figure 5. Yellow fluorescence intensity (x-axes) versus green fluorescence intensity (y-axes) for YFP single point mutants compared to the WT protein. We performed fluorescence measurements in three batches where each batch contained three replicate mutagenized libraries of mutants and that of wild-type (YFP) protein. We did this because the absolute measurements of fluorescence intensities are more sensitive to experimental error compared to correlation between the two intensities. Here, the three panels show the three batches we used in experiments. For each batch, we compared the mean yellow and green fluorescence intensities with those of wild-type YFP (red) in the same batch. Error bars on the plot represent one standard deviation in either green or yellow fluorescence intensity in three replicates (n=3).

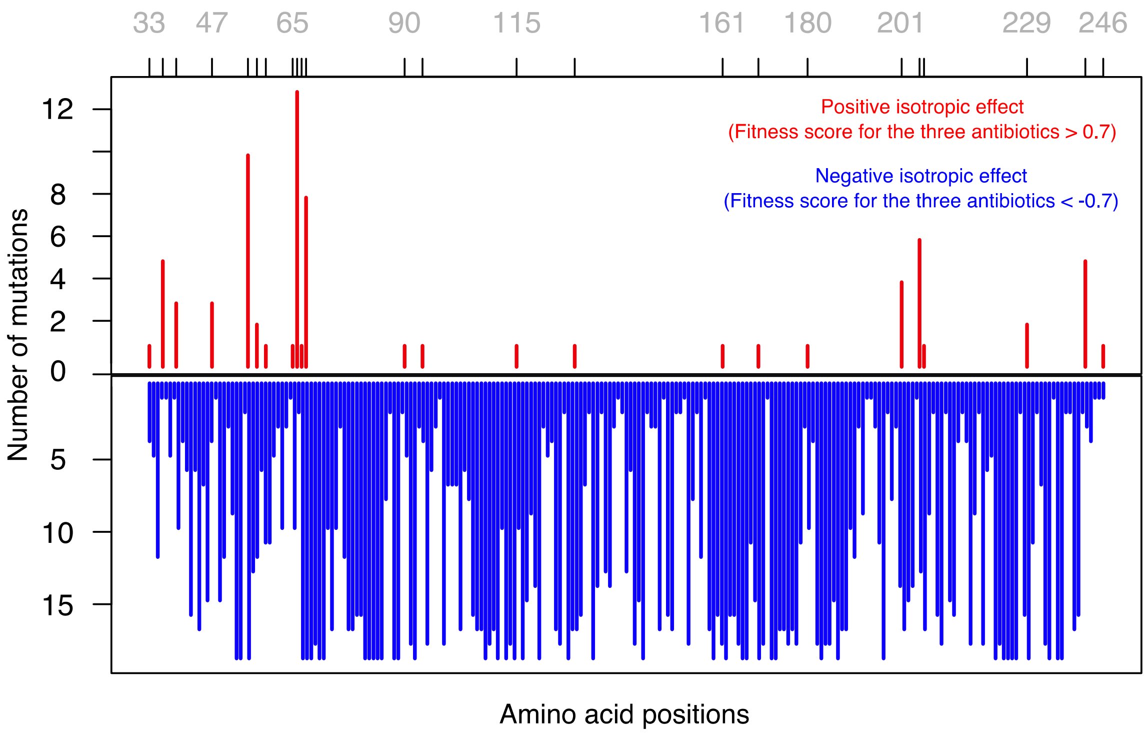

Supplementary Figure 6. The number of mutations at 240 amino acid positions of VIM2 with a significant positive effect (in red) and negative effect (in blue) on resistance to the antibiotics ampicillin, cefotaxime and meropenem. Data is taken from our previous deep mutational scan of VIM2[2]. We considered mutations for whom the fitness scores on all three antibiotics (ampicillin, cefotaxime, and meropenem) exceeded 0.7 as having a positive effect. Conversely, we considered mutations with fitness scores below -0.7 as having negative effects (Supplementary Note 3). The concentration of ampicillin, cefotaxime, and meropenem in these experiments were 128 μg/ml, 4 μg/ml, and 0.031μg/ml, respectively.

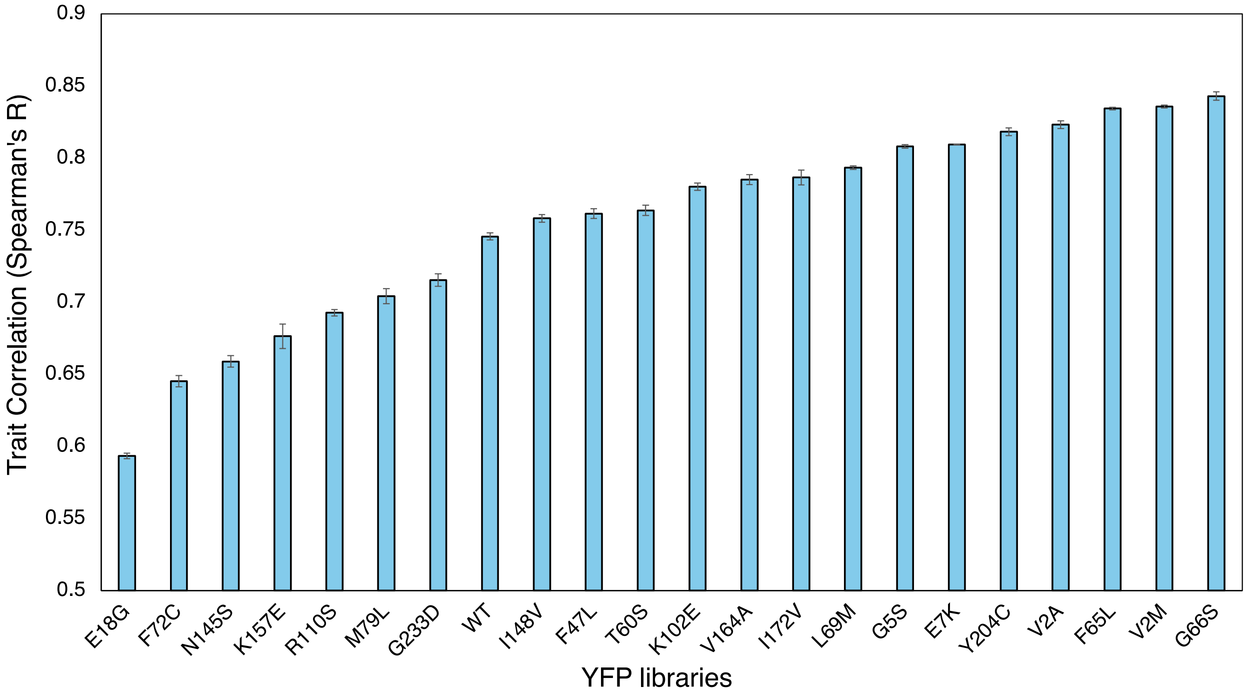

Supplementary Figure 7. Spearman's rank correlation between yellow and green fluorescence intensities of 21 YFP mutagenesis libraries. Each library contained DNA sequences resulting from the mutagenesis of a different YFP mutant (vertical axis) at a rate of approximately 0.8 amino acid mutations per YFP molecule[3]. Error bars on the plot represent one standard deviation in correlation coefficients in three replicate measurements. All correlations were highly significantly different from zero, with p-values < 10^{-16}. Changes in correlation coefficients from the mutagenesis library with the lowest correlation (E18G) to the one with the highest correlation (G66S) is significant using Fisher's Z-test ($p \sim 0$; Supplementary Note 4).

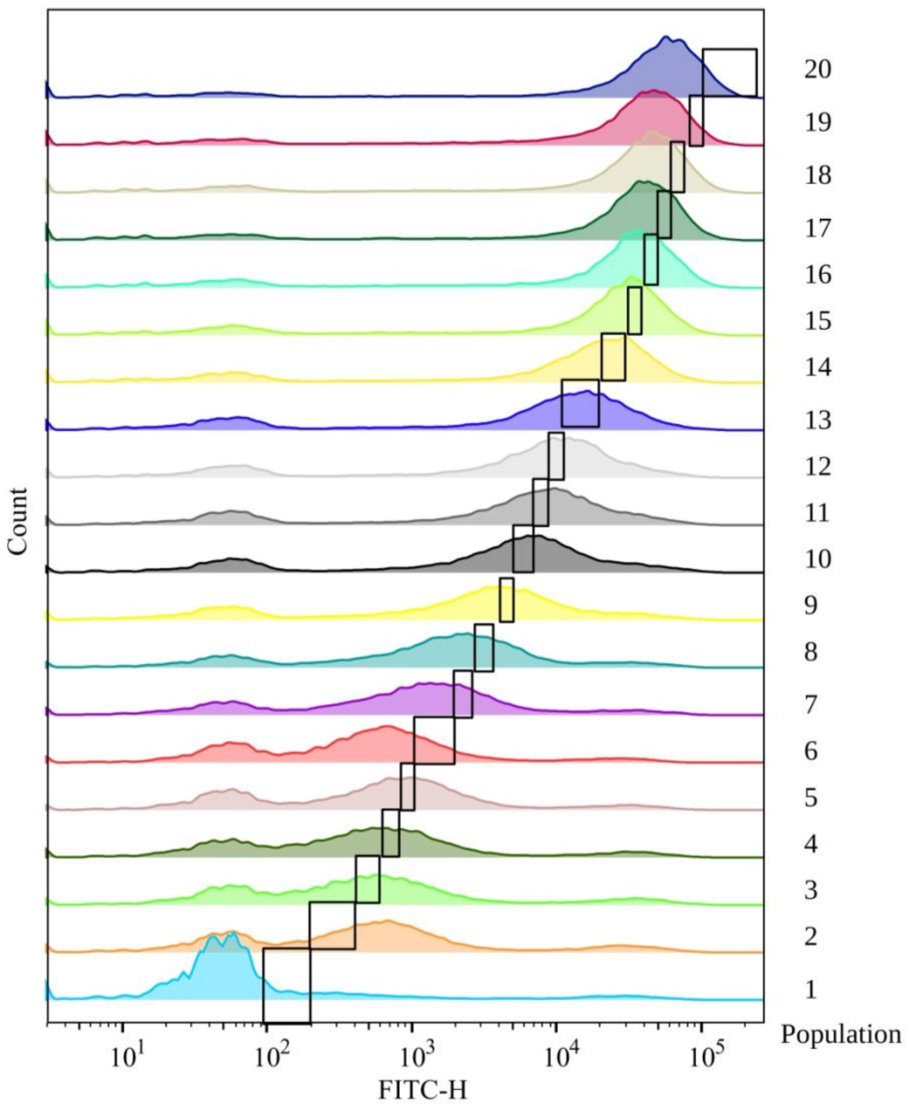

Supplementary Figure 8. Distribution of gates for re-sorting the cells in subpopulations 1-20 (see Methods). The vertical axis indicates the number of cells at a given value of yellow fluorescence intensity (arbitrary unit, the horizontal axis) in each population. Each of the subpanels, from bottom to top, represents the distribution of fluorescence intensities of cells in each subpopulation 1-20 (as indicated on the right side of the panel). We re-sorted each of these twenty populations by selecting cells whose yellow fluorescence intensities fall into the corresponding bin, which is highlighted in a rectangle.

Supplementary Table 1. The Spearman's rank correlation coefficient between green and yellow fluorescence intensities of YFP mutants. The symbols G, Y and U correspond to the mutations G66S, Y204C, and G66S-Y204C, respectively.

Mutation	R[a]	Mutation	R	Mutation	R
U_F72S	0.172	U_K141R	0.698	U_F65L	0.874
E18G	0.230	U_I129T	0.709	U_V2A	0.880
U_M79L	0.278	U_K167E	0.709	U_V2A	0.880
U_E18G	0.392	U_G5S	0.723	G_F65S	0.886
M79L	0.416	U_R74H	0.742	U_I168V	0.906
R110S	0.448	U_F65S	0.746	Y_V2M	0.923
U_R110S	0.460	U_K102R	0.753	Y_V2A	0.925
G5S	0.465	U_F47L	0.755	U_V2M	0.927
E7K	0.505	U_I172V	0.783	U_V2M	0.927
U_F65S	0.525	Y204C	0.784	G66S	0.929
K102E	0.525	U_V164A	0.789	G_F47L	0.929
WT	0.527	Y_N145S	0.809	G_F72I	0.930
K157E	0.535	Y_F72I	0.814	G_F72C	0.943
G233D	0.576	U_T60S	0.822	G_K141R	0.950
U_K157E	0.586	Y_V164A	0.832	G_V164A	0.951
U_G233D	0.616	Y_F47L	0.840	G_K102R	0.951
U_K42R	0.617	Y_K141R	0.842	G_V2A	0.954
U_E7K	0.628	Y_K102R	0.847	G_I172V	0.955
U_E91A	0.662	Y_I129T	0.848	G_F65L	0.956
U_I48V	0.671	Y_F72C	0.859	G_I168V	0.956
U	0.683	U_F72I	0.861	G_I129T	0.958
U_K102E	0.687	Y_I172V	0.864	G_V2M	0.960
U_N145S	0.689	U_F72C	0.865	G_N145S	0.982
Y_I168V	0.694	Y_F65L	0.871		

a: The Spearman's correlation coefficient between yellow and green fluorescent intensities.

Supplementary Table 2. Maximum emission wavelength (nm), refolding yield after thermal denaturation (%), and Spearman's rank correlation coefficient R between green and yellow fluorescence intensities of a selected set of 10 YFP mutants.

Mutant	Max. emission wavelength (nm)	Refolding yield (%)	Trait correlation
G66S	524	45.45	0.9285
WT	525	30.56	0.5271
Y204C	514	35.68	0.7844
K102E	530	37.84	0.5252
G66S-Y204C	510	32.5	0.6828
G66S-Y204C -F47L	512	49.66	0.7546
G66S-Y204C -F65L	511	61.5	0.8738
G66S-Y204C -V164A	511	54.01	0.7888
G66S-Y204C -I172V	511	41.27	0.7829
G66S-Y204C -K102E	510	36.67	0.6868

Supplementary Table 3. Results of a multiple linear regression analysis for the relationship between the overall trait correlation, R, and the trait correlations between green and yellow fluorescence intensities of cells whose yellow fluorescence are surpassing the indicated quantile (R_{high}) and those falling below this quantile (R_{low}).

lm ($R \sim R_{high} + R_{low}$)					
quantile		Estimate[a]	Std. Error[b]	t-value[c]	Pr (>|t|)[d]
10%	(Intercept)	0.03443	0.01398	2.463	0.0163
	R_{high}	0.97906	0.02609	37.52	2x10-16
	R_{low}	0.01375	0.02181	0.63	0.5306
20%	(Intercept)	0.09367	0.019	4.93	5.44x10-6
	R_{high}	0.84997	0.03695	23.003	< 2x10-16
	R_{low}	0.13367	0.02825	4.732	1.14x10-5
50%	(Intercept)	0.25065	0.02511	9.981	5.04x10-15
	R_{high}	0.57882	0.06310	9.174	1.44x10-13
	R_{low}	0.25397	0.04625	5.491	6.23x10-07

a: the estimated coefficient for the predictor variable in the regression equation. b: the standard error of the coefficient estimate. c: the t-statistic, for the test of the null hypothesis that the coefficient estimate is not significantly different from zero. d: the p-value associated with the t-value. It indicates the probability of observing a t-value as extreme as the one obtained, assuming the null hypothesis is true. The correlation coefficients (R_{high}, and R_{low}) mentioned are Spearman's correlation coefficients.

Supplementary Table 4. The number of mutations that significantly increase resistance (positive effect, score > 0.7), decrease resistance (negative effects, score < -0.7) or are neutral (-0.7 ≤ score ≤ 0.7).

Antibiotic(s)	Positive Effect (Score ≥ 0.7)	Negative Effect (Score ≤ -0.7)	Neutral (-0.7 < Score| < 0.7)
AMP[a]	131	3074	1360
CTX[a]	146	2819	1600
MEM[a]	140	2925	1500
AMP & CTX	96	2778	1691
AMP & MEM	87	2871	1607
CTX & MEM	94	2718	1753
AMP & CTX & MEM	75	2073	2417

a: The concentrations of ampicillin, cefotaxime, and meropenem were 128 μg/ml, 4 μg/ml, and 0.031μg/ml, respectively.

Supplementary Table 5. P-values of Wilcoxon's rank sum tests asking whether average yellow and average green fluorescence intensities of various single point mutations (left-most column) differ from the corresponding fluorescence intensities of wild-type YFP. Entries highlighted in green indicate mutants with both yellow and green fluorescence intensities significantly higher than those of wild-type YFP. Entries highlighted in orange correspond to mutants with yellow and green fluorescence intensities significantly higher than those of wild-type YFP. The variants G66S and Y204C are highlighted in blue. These variants exhibit a higher green fluorescence intensity but a lower yellow fluorescence intensity compared to the wild-type YFP. We calculated all p-values using one-sided Wilcoxon's rank sum tests, using the alternative hypothesis of higher or lower fluorescence intensities as indicated in the first row. All pertinent measurements were performed in triplicate.

Mutants	p_{Yellow}(>WT)[a]	p_{Green}(>WT)[b]	p_{Yellow}(<WT)[c]	p_{Green}(<WT)[d]	Isotropic
F47L	0.00454545	0.98181818	1	0.03181818	*NO*
F65L	0.00454545	0.5738587	1	0.5	*NO*
G66S	1	0.00454545	0.00454545	1	*NO*
I172V	0.00454545	0.00774558	1	0.99543163	*YES*
K102E	0.00454545	0.04746465	1	0.96823756	*YES*
V164A	0.00454545	0.00454545	1	1	*YES*
V2A	0.00454545	0.00454545	1	1	*YES*
V2M	0.00454545	0.00454545	1	1	*YES*
Y204C	1	0.00454545	0.00454545	1	*NO*
E18G	1	0.99530747	0.00454545	0.00792938	*YES*
E7K	0.03181818	0.93080235	0.98181818	0.09738973	*NO*
F72C	1	0.99524477	0.00454545	0.00802198	*YES*
G5S	0.00454545	0.03897346	1	0.97430895	*NO*
I148V	0.85909091	0.90261027	0.18636364	0.13320994	*NO*
L69M	1	0.95224454	0.00454545	0.06919765	*NO*
M79L	1	0.99530747	0.00454545	0.00792938	*YES*
R110S	1	0.99530747	0.00454545	0.00792938	*YES*
T60S	1	0.99530747	0.00454545	0.00792938	*YES*
G233D	0.81363636	0.82280841	0.24090909	0.22937805	*NO*
K157E	0.96818182	0.96076468	0.05	0.05769688	*NO*
N145S	0.96818182	0.99379955	0.05	0.0102982	*NO*

p-values calculated from Wilcoxon's rank sum test for a: The average yellow fluorescence intensity of the mutant is higher than that of wild-type YFP, b: The average green fluorescence intensity of the mutant is higher than that of wild-type YFP, c: The average yellow fluorescence intensity of the mutant is lower than that of wild-type YFP, and d: The average green fluorescence intensity of the mutant is higher than that of wild-type YFP.

Supplementary Table 6. Ampicillin Minimum Inhibitory Concentration (µg/mL) of different VIM2 variants sampled from each round of the evolution experiment.

Round	Number of values	Minimum	Maximum	Range	Mean	Fold-Range	Std. Deviation	Std. Error of Mean
1	24	64	16384	16320	7299	256	5914	1207
2	24	64	16384	16320	4504	256	4667	953
5	24	28	8192	8164	1371	293	2465	503
10	48	8	4096	4088	543	512	943	136
15	48	8	4096	4088	459	512	987	143
20	48	16	4096	4080	443	256	726	105
30	23	16	2048	2032	595	128	616	128
40	72	8	2048	2040	247	256	334	39
50	48	4	2048	2044	155	512	325	47
60	92	4	2048	2044	274	512	314	33
70	47	128	1024	896	387	8	248	36
80	71	8	2048	2040	362	256	379	45
90	48	32	1024	992	393	32	294	42
100	168	64	4096	4032	776	64	685	53

Supplementary Table 7. Meropenem Minimum Inhibitory Concentration (µg/mL) of different VIM2 variants sampled from each round of the evolution experiment.

Round	Number of values	Minimum	Maximum	Range	Mean	Fold-Range	Std. Deviation	Std. Error of Mean
1	24	0.032	8	8	3.70	250	3.2	0.660
2	24	0.063	8	7.9	1.80	127	2.4	0.500
5	24	0.016	8	8	0.64	500	1.8	0.370
10	48	0.016	1	512	11.00	63	74	11.000
15	48	0.016	2	2	0.17	125	0.35	0.050
20	48	0.016	1	0.98	0.14	63	0.22	0.031
30	23	0.016	1	0.98	0.26	63	0.32	0.068
40	72	0.013	0.5	0.49	0.08	38	0.098	0.012
50	48	0.013	1	0.99	0.08	77	0.16	0.023
60	91	0.016	0.5	0.48	0.08	31	0.094	0.010
70	23	0.25	8	7.8	1.90	32	1.9	0.400
80	72	0.016	0.5	0.48	0.09	31	0.11	0.013
90	24	0.016	0.25	0.23	0.08	16	0.074	0.015
100	93	0.016	4	4	0.43	1	0.057	0.006

Supplementary Table 8. Cefotaxime Minimum Inhibitory Concentration (µg/mL) of different VIM2 variants sampled from each round of the evolution experiment.

Round	Number of values	Minimum	Maximum	Range	Mean	Fold-Range	Std. Deviation	Std. Error of Mean
1	24	2	512	510	224	256	185	38
2	24	1	512	511	149	512	150	31
5	24	0.25	256	256	35	1024	70	14
10	48	0.25	512	512	26	2048	81	12
15	48	0.25	128	128	14	512	31	4.5
20	48	0.13	128	128	11	985	24	3.4
30	23	0.25	128	128	18	512	30	6.2
40	72	0.063	256	256	8	4063	32	3.8
50	48	0.063	16	16	2.5	254	4.5	0.65
60	91	0.063	16	16	2.9	254	4.2	0.45
70	23	0.032	0.5	0.47	0.12	16	0.15	0.032
80	72	0.13	16	16	1.3	123	2.3	0.27
90	24	0.13	4	3.9	0.89	31	0.95	0.19
100	93	0.13	64	64	4.4	492	9.2	0.96

Supplementary Note 1

In this analysis we aimed to differentiate between changes in trait correlation that arise from changes in the genetic background, i.e., they are properties of specific mutants, or simply from changes in the fraction of cells with weak or no fluorescence. Specifically, and for an isogenic population of each mutant, we calculated two correlations between the yellow and green fluorescence intensities. The first correlation was between these intensities for cells whose yellow fluorescent fell below the 50% of the overall population's fluorescence (denoted as R_{low}). The second correlation was between these intensities for cells whose yellow fluorescent fell above the 50% of the overall population's fluorescence (denoted as R_{high}). We repeated these calculations with the percentile of 20% and 10% as well. As sown in Table S3, we find that for each mutant population and for the three percentiles (10%, 20%, 50%), R_{high} has a more significant impact on the overall trait correlation than R_{low} ($p \sim 10^{-16}$; multiple linear regression models). This suggests that the changes in trait correlation among genotypes stem from variations in the fluorescence intensity of functional and actively fluorescing molecules.

Supplementary Note 2

In VIM2 populations and for ampicillin and cefotaxime resistance, we further investigated whether resistance level might be a confounding factor in the relationship between trait correlation and predicted folding stability. Specifically, populations with lower resistance against ampicillin may exhibit a weaker resistance correlation independent of protein folding stability. To find out, we formulated two nested linear models by incorporating both stability and resistance level as predictors of resistance correlation. We then conducted likelihood ratio tests to assess the significance of the gain in likelihood upon adding either protein stability or resistance to the predictive model. The likelihood only changed when we added protein stability as a second predictor (p~0.005; likelihood ratio test with 1 degree of freedom), but not the resistance level (p~0.22; likelihood ratio test with 1 degree of freedom). We therefore conclude that changes in protein folding drive the correlation between ampicillin and cefotaxime resistance in our evolving VIM2 population.

Supplementary Note 3

We estimated the isotropic effect of mutations in VIM2 using our previous deep mutational scan of VIM2[2]. To categorize these mutations as either deleterious, beneficial, or neutral, we applied a fitness score threshold of 0.7. We had previously estimated this threshold through a Z-test, where we compared the fitness scores of each variant to the fitness distribution of 244 synonymous variants (representing the null model distribution), adjusting for a 5% false discovery rate using the Benjamini-Hochberg procedure[2]. We then classified mutations into three groups: those associated with a negative impact on fitness (fitness score < −0.7), those resulting in no fitness effects (fitness score ranging from -0.7 to 0.7), and those with a positive fitness effect (fitness score > 0.7). We define an isotropic mutation as one whose fitness score lies below -0.7 or above 0.7, on all three antibiotics (Table S4).

Supplementary Note 4

To find out whether a change in correlation between two quantities is significant, we used Fisher's Z-test. To this end, we first converted two Spearman correlation coefficients R_1 and R_2 into the z-scores z_1 and z_2:

$$z_1 = 0.5 \ln\left(\frac{1+R_1}{1-R_1}\right) \qquad \text{(Eq. 1)}$$

$$z_2 = 0.5 \ln\left(\frac{1+R_2}{1-R_2}\right) \qquad \text{(Eq. 2)}$$

We then calculated the standard errors of the z-scores, s_1 and s_2, as

$$s_1 = \frac{1}{\sqrt{N_1-2}} \qquad \text{(Eq. 3)}$$

$$s_2 = \frac{1}{\sqrt{N_2-2}} \qquad \text{(Eq. 4)}$$

In equations 3 and 4, we subtracted 2 from the sample size because two degrees of freedom are lost when we estimate population parameters from the sample, one for each of the two variables involved. We then calculated the test statistic Z using the following formula:

$$Z = \frac{z_1-z_2}{\sqrt{{s_1}^2+{s_2}^2}} \qquad \text{(Eq. 5)}$$

and determined the corresponding p-value as:

$$p = 2 * (1 - pnorm(|Z|) \qquad \text{(Eq. 6)}$$

Here, $pnorm(|Z|)$ represents the cumulative distribution function (CDF) of the standard normal distribution (with mean zero and variance 1) for the absolute value |Z| of the test statistic, Z.

References:

1. Chen, J., Fowler, D. & Tokuriki, N. Environmental selection and epistasis in an empirical phenotype–environment–fitness landscape. *Nature Ecology & Evolution* **6**, 427-438 (2022).
2. Chen, J.Z., Fowler, D.M. & Tokuriki, N. Comprehensive exploration of the translocation, stability and substrate recognition requirements in VIM-2 lactamase. *Elife* **9**, e56707 (2020).
3. Zheng, J., Guo, N. & Wagner, A. Selection enhances protein evolvability by increasing mutational robustness and foldability. *Science* **370**(2020).
