# Supplementary material for: Rapid evolutionary change in trait correlations of single proteins
Source: Nat Commun. 2024 Apr 18;15:3327. doi: 10.1038/s41467-024-46658-1 (PMC11026499; doi:10.1038/s41467-024-46658-1)
Supplement: Supplementary file 3 — Reporting Summary [file 41467_2024_46658_MOESM3_ESM.pdf]

Corresponding author(s): Andreas Wagner, and Pouria Dasmeh

Last updated by author(s): Feb 19, 2024

Reporting Summary

Nature Portfolio wishes to improve the reproducibility of the work that we publish. This form provides structure for consistency and transparency in reporting. For further information on Nature Portfolio policies, see our Editorial Policies and the Editorial Policy Checklist.

Statistics

For all statistical analyses, confirm that the following items are present in the figure legend, table legend, main text, or Methods section.

n/a	Confirmed	
☐	☒	The exact sample size (*n*) for each experimental group/condition, given as a discrete number and unit of measurement
☐	☒	A statement on whether measurements were taken from distinct samples or whether the same sample was measured repeatedly
☐	☒	The statistical test(s) used AND whether they are one- or two-sided<br>*Only common tests should be described solely by name; describe more complex techniques in the Methods section.*
☒	☐	A description of all covariates tested
☒	☐	A description of any assumptions or corrections, such as tests of normality and adjustment for multiple comparisons
☐	☒	A full description of the statistical parameters including central tendency (e.g. means) or other basic estimates (e.g. regression coefficient) AND variation (e.g. standard deviation) or associated estimates of uncertainty (e.g. confidence intervals)
☐	☒	For null hypothesis testing, the test statistic (e.g. *F*, *t*, *r*) with confidence intervals, effect sizes, degrees of freedom and *P* value noted<br>*Give P values as exact values whenever suitable.*
☒	☐	For Bayesian analysis, information on the choice of priors and Markov chain Monte Carlo settings
☒	☐	For hierarchical and complex designs, identification of the appropriate level for tests and full reporting of outcomes
☐	☒	Estimates of effect sizes (e.g. Cohen's *d*, Pearson's *r*), indicating how they were calculated

Our web collection on statistics for biologists contains articles on many of the points above.

Software and code

Policy information about availability of computer code

Data collection	We used Fortessa cell analyzer (BD Biosciences): for collecting fluorescence intensity data of evolving populations and engineered variants.<br>We used Tecan Infinite F200 microplate reader / Tecan Spark multimode microplate reader for collecting fluorescence emission spectra data of engineered variants.
Data analysis	We used the following softwares with their corresponding versions: R v4.2.1; Python 3.10.4; SMRT Link V9.0.0.92188; FlowJo V10.4.2 & 10.8.1<br><br>Scripts, raw and processed data, and statistical analyses are available at: https://github.com/dasmeh/Trait_correlation

For manuscripts utilizing custom algorithms or software that are central to the research but not yet described in published literature, software must be made available to editors and reviewers. We strongly encourage code deposition in a community repository (e.g. GitHub). See the Nature Portfolio guidelines for submitting code & software for further information.

Data

Policy information about availability of data

All manuscripts must include a data availability statement. This statement should provide the following information, where applicable:

- Accession codes, unique identifiers, or web links for publicly available datasets
- A description of any restrictions on data availability
- For clinical datasets or third party data, please ensure that the statement adheres to our policy

We used the 3D structure of VIM2 with the pdb id=4BZ3 (https://www.rcsb.org/structure/4BZ3) for structure visualization. We used previously published deep mutational scanning data of VIM2 for trait correlation analyses performed on mutational data (raw data available at: BioProject ID PRJNA606894, processed data available at: https://cdn.elifesciences.org/articles/56707/elife-56707-supp2-v2.xlsx)

Research involving human participants, their data, or biological material

Policy information about studies with human participants or human data. See also policy information about sex, gender (identity/presentation), and sexual orientation and race, ethnicity and racism.

Reporting on sex and gender	NA
Reporting on race, ethnicity, or other socially relevant groupings	NA
Population characteristics	NA
Recruitment	NA
Ethics oversight	NA

Note that full information on the approval of the study protocol must also be provided in the manuscript.

Field-specific reporting

Please select the one below that is the best fit for your research. If you are not sure, read the appropriate sections before making your selection.

☐ Life sciences ☐ Behavioural & social sciences ☒ Ecological, evolutionary & environmental sciences

For a reference copy of the document with all sections, see nature.com/documents/nr-reporting-summary-flat.pdf

Ecological, evolutionary & environmental sciences study design

All studies must disclose on these points even when the disclosure is negative.

Study description	To study how trait correlation evolves through mutation and selection, we subjected populations of yellow fluorescent protein (YFP) and VIM2 metallo-beta-lactamase to directed evolution under different selection strengths. We then quantify how mutations and selection shape the correlations between two traits in the fluorescent protein populations (the ability to emit yellow and green light) and between three traits in our enzyme populations (the resistance against ampicillin, cefotaxime, and meropenem). Through engineering mutants and biochemical assays, we revealed that mutation and selection can drive the evolution of trait correlation by shaping protein foldability (a protein's ability to fold).
Research sample	pBAD202/D-TOPO (K4202-01, Invitrogen); E. coli strain BW27783 (CGSC 12119); E.cloni® 10G E. coli cells (Lucigen Corp.)<br>We used the vector pBAD202/D-TOPO (K4202-01, Invitrogen), which carries an arabinose-inducible araBAD promoter and a Kanamycin-resistance gene for YFP evolution within the E. coli strain BW27783 (CGSC 12119). For antibiotic resistance experiments, we cloned the VIM2-coding gene into a low-copy number plasmid with a constitutive, low expression TEM promoter and chloramphenicol resistance. These combination are optimal for our selection assays as we have previously demonstrated (Science 370.6521 (2020): eabb5962., Elife 9 (2020): e56707.). Both E.coli strains are highly efficient electrocompetent cells.
Sampling strategy	We did not perform a statistical test to pre-determine the sample size. However, we ensured an appropriate number of biological replicates based on the magnitude and consistency of measurable differences between groups. The sample sizes for all measurements are described in the figure legends.
Data collection	We performed data collection using computer softwares. We used excel sheets to record fluorescent data. We used .fcs files to record the output of FACS library sorting. We used pen and paper to write down the number of counted colonies.
Timing and spatial scale	Data collection for AMP, CTX and MEM MICs happened intermittently between 06'2018-08'2021, with a frequency of once every 1-2 months based on when the mutant libraries were generated. We kept the number of colonies on the plate used for selection to 20,000, and sampled between 24-96 variants from every 5-10 libraries. There was a pause in data collected between 01'-05'2019 and 09'2019-08'2020 for attending courses. Data collection for YFP was conducted from 2016 to 2021 and from 2018 to 2023 respectively. Jia Zheng and Pouria Dasmeh recorded the data for YFP experiments. Ayse Erdogan recorded the data from VIM2

	populations.
Data exclusions	No samples were excluded from the analysis.
Reproducibility	All measurements were performed at least in three biological replicates.
Randomization	We did not perform randomization as this procedure is not relevant for our study. We selected all surviving colonies for our VIM2 experiments and hence did not require a randomized selection. Also, the FACS experiments generate cells that are all sorted into different bins. We selected all the bins in these experiments without the need to randomization.
Blinding	This procedure is not relevant for our study.As the surviving colonies carrying different enzyme variants after ampicillin selection had similar colony sizes, we had no way of pre-assessing the resistance strength of colonies picked for AMP/CTX/MEM MIC characterization, thus the process of picking colonies from the plate was randomized and did not need blinding to the conditions.

Did the study involve field work? ☐ Yes ☒ No

Reporting for specific materials, systems and methods

We require information from authors about some types of materials, experimental systems and methods used in many studies. Here, indicate whether each material, system or method listed is relevant to your study. If you are not sure if a list item applies to your research, read the appropriate section before selecting a response.

Materials & experimental systems

n/a	Involved in the study	
☐	☒	Antibodies
☒	☐	Eukaryotic cell lines
☒	☐	Palaeontology and archaeology
☒	☐	Animals and other organisms
☒	☐	Clinical data
☒	☐	Dual use research of concern
☒	☐	Plants

Methods

n/a	Involved in the study	
☒	☐	ChIP-seq
☐	☒	Flow cytometry
☒	☐	MRI-based neuroimaging

Antibodies

Antibodies used	The quantification of soluble fluorescent proteins in evolving populations was performed using a GFP ELISA Kit (AKR-121, Cell Biolabs Inc.).
Validation	For the GFP ELISA Kit, please refer to https://www.cellbiolabs.com/sites/default/files/AKR-121-gfp-elisa-kit.pdf

Plants

Seed stocks	NA
Novel plant genotypes	NA
Authentication	NA

Flow Cytometry

Plots

Confirm that:

- [x] The axis labels state the marker and fluorochrome used (e.g. CD4-FITC).
- [x] The axis scales are clearly visible. Include numbers along axes only for bottom left plot of group (a 'group' is an analysis of identical markers).
- [x] All plots are contour plots with outliers or pseudocolor plots.
- [x] A numerical value for number of cells or percentage (with statistics) is provided.

Methodology

Sample preparation	The details of sample preparation are added to Methods, sections "Sorting cells from a pool of evolving populations at the end of directed evolution" and "Fluorescence assay using flow cytometry".
Instrument	FACSAria III 4L (BD);LSR II Fortessa (BD)
Software	BD FACSDIVA software; FlowJo V10.4.2 & 10.8.1 (LLC)
Cell population abundance	We selected cells by their yellow fluorescence intensity according to the selection criteria described in Figures 2A-B with an Aria III cell sorter (BD Biosciences).
Gating strategy	We sorted cells at 4 °C in the FITC channel (λex = 488 nm, λem = 530±15 nm), and collected 105 cells in ~1 ml LB medium for each sorted subpopulation. The gating strategy is explained in Methods and shown in Figure S8.

- [x] Tick this box to confirm that a figure exemplifying the gating strategy is provided in the Supplementary Information.
